# Dietary methionine restriction alleviates oxidative stress and inflammatory responses in lipopolysaccharide-challenged broilers at early age

**DOI:** 10.3389/fphar.2023.1120718

**Published:** 2023-02-15

**Authors:** Xiyuan Pang, Zhiqiang Miao, Yuanyang Dong, Huiyu Cheng, Xiangqi Xin, Yuan Wu, Miaomiao Han, Yuan Su, Jianmin Yuan, Yuxin Shao, Lei Yan, Jianhui Li

**Affiliations:** ^1^ College of Animal Sciences, Shanxi Agricultural University, Taigu, China; ^2^ College of Animal Sciences and Technology, China Agricultural University, Beijing, China; ^3^ Institute of Animal Husbandry and Veterinary Medicine, Beijing Academy of Agriculture and Forestry Sciences, Beijing, China; ^4^ New Hope Liuhe Co.,Ltd., Beijing, China

**Keywords:** methionine restriction, lipopolysaccharide, antioxidant capacity, inflammation, liver health, broilers

## Abstract

In this study, we investigated the effect of dietary methionine restriction (MR) on the antioxidant function and inflammatory responses in lipopolysaccharide (LPS)-challenged broilers reared at high stocking density. A total of 504 one-day-old male Arbor Acre broiler chickens were randomly divided into four treatments: 1) CON group, broilers fed a basal diet; 2) LPS group, LPS-challenged broilers fed a basal diet; 3) MR1 group, LPS-challenged broilers fed a methionine-restricted diet (0.3% methionine); and 4) MR2 group, LPS-challenged broilers fed a methionine-restricted diet (0.4% methionine). LPS-challenged broilers were intraperitoneally injected with 1 mg/kg body weight (BW) of LPS at 17, 19, and 21 days of age, whereas the CON group was injected with sterile saline. The results showed that: LPS significantly increased the liver histopathological score (*p* < 0.05); LPS significantly decreased the serum total antioxidant capacity (T-AOC), superoxide dismutase (SOD), and glutathione peroxidase (GSH-Px) activity at 3 h after injection (*p* < 0.05); the LPS group had a higher content of Interleukin (IL)-1β, IL-6, and tumor necrosis factor-α (TNF)-α, but a lower content of IL-10 than the CON group in serum (*p* < 0.05). Compared with the LPS group, the MR1 diet increased catalase (CAT), SOD, and T-AOC, and the MR2 diet increased SOD and T-AOC at 3 h after injection in serum (*p* < 0.05). Only MR2 group displayed a significantly decreased liver histopathological score (*p* < 0.05) at 3 h, while MR1 and MR2 groups did so at 8 h. Both MR diets significantly decreased serum LPS, CORT, IL-1β, IL-6, and TNF-α contents, but increased IL-10 content (*p* < 0.05). Moreover, the MR1 group displayed significantly increased expression of nuclear factor erythroid 2-related factor 2 (*Nrf2*), *CAT*, and *GSH-Px* at 3 h; the MR2 group had a higher expression of Kelch-like ECH-associated protein 1 (*Keap1*), *SOD*, and *GSH-Px* at 8 h (*p* < 0.05). In summary, MR can improve antioxidant capacity, immunological stress, and liver health in LPS-challenged broilers. The MR1 and MR2 groups experienced similar effects on relieving stress; however, MR1 alleviated oxidative stress more rapidly. It is suggested that precise regulation of methionine levels in poultry with stress may improve the immunity of broilers, reduce feed production costs, and increase production efficiency in the poultry industry.

## Introduction

Bacterial diseases are an increasingly severe problem that restricts the development of the poultry industry. With increased stocking density in intensive production, the immune competence of birds decreases, making the body much more susceptible to bacterial diseases (Averós and Estevez, 2018; Goo et al., 2019). Lipopolysaccharide (LPS) is one of the main pathogenic factors of bacterial diseases in poultry. It is a component of the cell wall of Gram-negative bacteria, which can reduce the antioxidant capacity of poultry, cause inflammation, and produce inflammatory cytokines. (Leshchinsky and Klasing, 2001; Takahashi et al., 2011). After infection, LPS molecules in pathogens are recognized and regulated by lipopolysaccharide binding protein (LBP)/CD14 in the cell membrane to form the LPS-LBP complex, which binds to CD14/TLRs receptors on the cell surface to induce tumor necrosis factor (TNF)-α, interleukin (IL)-1β, and other cytokines, resulting in inflammation (Bryant et al., 2010). In addition, LPS activates phospholipase C (PLC) and releases diglycerol and inositol 1, 4, 5-Trisphosphate (IP3) in Kupffer’s cells. Inositol 1, 4, 5-Trisphosphate induces an increase in intracellular Ca^2+^ concentration. The increase in intracellular Ca^2+^ increases the activity of reactive oxygen species (ROS)-producing enzymes and accelerates the synthesis of free radicals through the mitochondrial respiratory chain, causing an oxidative stress response (Jin et al., 2006). It has been reported that LPS challenge reduces the antioxidant level and antioxidant enzyme activity and improves the level of inflammatory factors such as IL-1β and IL-6 in the serum of broilers (Zheng et al., 2016; Zheng et al., 2020). In addition, *in vitro* studies reported that excessive accumulation of inflammatory cytokines induced by LPS can damage pig intestinal epithelial cells and lead to intestinal dysfunction (Li et al., 2022). Therefore, alleviating the inflammatory response and oxidative stress caused by avian bacterial diseases is an urgent problem in the feeding industry.

Methionine (MET), the first restricted amino acid in broilers, plays a crucial role in regulating the poultry’s growth and development, antioxidant capacity, and immune function (Xie et al., 2007). However, recent studies have discovered that reducing the dietary methionine level, known as methionine restriction (MR), can also improve the anti-inflammatory and antioxidant capacities of animals (Fang et al., 2022). It has been reported that proper MR (the methionine level in the diet is 60%–80% of the NRC level) can significantly improve intestinal antioxidant capacity, gene expression of the intestinal innate immune system, and intestinal flora structure of broilers (Yu, 2013). MR has been shown to reduce the plasma concentrations of LPS and LBP and the mRNA expression of ileal TNF-α and IL-6 genes in mice fed a high-fat diet, indicating that MR could reduce the inflammatory response by limiting the expression of inflammatory factors (Wu et al., 2020a). Further, MR activates nuclear factor erythroid 2-related factor 2 (*Nrf2*), and the increased activity of *Nrf2* and the enhanced expression of the accompanying genes related to the antioxidant response will improve the antioxidant and detoxification abilities of the body (Brown-Borg and Buffenstein, 2017). In addition, MR alleviates the infiltration of inflammatory factors and tissue damage in LPS-challenged mice by promoting hydrogen sulfide production (Duan et al., 2022). However, whether MR can protect broiler chicks against LPS-induced stress has not been reported.

Based on these findings, a double-stress model was used to study the effect of MR on the growth performance, liver antioxidant function, and inflammatory response in LPS-induced stress in young broilers reared at high stocking density. Our findings will help further optimize the methionine nutrition scheme of broilers infected with bacterial diseases under high-density feeding conditions and provide useful insights for alleviating production stress in broilers.

## Materials and methods

### Reagents, animals, and experimental design

LPS (*Escherichia coli* 055: B5) was obtained from Sigma Aldrich (St. Louis, MO, United States) and reconstituted in saline at a dose of 1.0 mg/mL.

A total of 504 one-day-old male Arbor Acre broiler chickens were randomly assigned to four treatments (six replicates of 21 birds per cage) in a completely randomized design. The experimental diets consisted of 1) Control group with a basal diet (CON), 2) LPS challenged group with basal diet (LPS), 3) LPS challenged group with a 0.3% methionine restriction diet (MR1), and 4) LPS challenged group with a 0.4% methionine restriction diet (MR2). All broilers were reared under high stocking density (30 birds/m^2^) in 0.7 m^2^ pens. At 17, 19, and 21 days, the LPS-challenged groups were intraperitoneally injected with 1 mg/kg body weight (BW) LPS solution, whereas the CON group received an equivalent volume of sterile saline injection (0.9%).

The birds were provided access to mash feed and water *ad libitum* during the 21 days of the experiment. For the first 3 days, the chicken house was kept under 24 h of continuous light, followed by 20 h of continuous light and 4 h of darkness from day 4 to day 21. The temperature was maintained at 33°C from days 1–3, and was gradually decreased by 2°C–3°C every week until it reached at 26°C and then was kept constant. In addition, all birds were vaccinated according to a routine immunization program. The present study was approved by the Animal Health and Care Committee of the Shanxi Agricultural University (Shanxi, China) and conducted according to the Guidelines for the Experimental Animal Welfare of Ministry of Science Technology of China (approval code. SXAU-EAW-2022Po.SD.01129001).

### Sample collection

On days 1, 17, and 21 of the experiment, the BW and total feed consumption of broilers were recorded to calculate average daily feed intake (ADFI), average daily gain (ADG), and feed conversion ratio (FCR) before (from 1 to 16 days of age) and after (from 17 to 21 days of age) the LPS challenge. The FCR = ADFI: ADG.

At 21 days of age, one bird from each replicate was selected for slaughter 3 and 8 h after LPS injection. Blood samples from the vein under the wing were collected into 5 mL anticoagulant-free vacutainer tubes. After centrifugation at 3,000 × *g* for 10 min at 4°C, the serum in the tube was collected and stored at −80°Cfor analysis. After blood sampling, broilers were euthanized by cervical dislocation and exsanguination for dissection of the liver, spleen, bursa, and thymus. The liver tissues were flash-frozen in liquid nitrogen and then preserved at −80°C for further analysis.

### Formulation of basal diets

The basal diet met the National Research Council (1994) nutrient requirements for broilers. The ingredient composition and nutrient levels are listed in Table 1.

**TABLE 1:** Composition and nutrient levels of basal diets (air-dry basis).

Items	Contents, %
Ingredients	
Corn	54.70
Soybean meal	33.50
Corn protein powder	3.00
Flour	2.00
Soybean Oil	2.40
Dicalcium phosphate	1.40
Limestone	1.35
Lys	0.38
NaCl	0.30
50% Choline chloride	0.20
Thr	0.11
98.5% arginine	0.04
Trace mineral^1^	0.20
Vitamin permmix^2^	0.03
DL-Met(99%)	0.20
Phytase	0.01
Zeolite powder	0.22
Total	100.00
Cacµgated nutrient level^3^	
ME (Kcal/kg)	2979
CP	21.80
Ca	0.90
Available phosPhorus	0.35
Total phosPhorus	0.67
Lys	1.24
Met	0.50
Lys+Cys	0.71
Thr	0.91
Trp	0.23

^1^ The mineral premix provided the following per kg of diets: Cu 8 mg; Zn 75 mg; Fe 80 mg; Mn 100 mg; Se 0.15 mg; I 0.35 mg.

^2^ The vitamin premix provided the following per kg of diets: Vitamin A 15000 TU; Vitamin D3 3600 TU; Vitamin K3 mg; Vitamin B1 2.4 mg; Vitamin B2 9.6 mg; Vitamin B6 3.6 mg; Vitamin B12 0.03 mg; Vitamin E 30 TU; biotin 0.15 mg; Folic acid 1.5 mg; Pantothenic acid 13.8 mg; Niacin 45 mg.

^3^ Nutrient levels were calculated values.

### Organ index

The organ indices of the liver, spleen, bursa, and thymus were calculated using the following equation (Deng et al., 2022)

$$organ\;index=organ\;weight\;\left(g\right)÷live\;body\;weight\;\left(kg\right)$$

### Serum biochemistry

The serum levels of aspartate aminotransferase (AST), alanine aminotransferase (ALT), γ-glutamyl transpeptidase (γ-GT), total protein (TP), and the albumin to globulin ratio (A:G) were quantified using a chemistry analyzer BS-800 (Shenzhen Mindray Bio-medical Electronics Co., Ltd., Shenzhen, China).

### Liver histology analysis

The liver of each bird was harvested, stored in 10% buffered formalin, and embedded in paraffin. For histological investigations, 3 μm sections were cut, deparaffinized, dehydrated, and stained with hematoxylin and eosin (H&E) (Ma et al., 2022). The tissue sections were observed for inflammatory changes under a light microscope, Leica DM 2500 M (Leica Microsystems, Wetzlar, Germany).

Liver tissues were observed for focal inflammation, mononuclear infiltration, loss of normal architecture, and necrosis. Acute liver injury was assessed using a composite inflammation and necrosis score as described by Siegmund et al. (2002). Lobular inflammation (0, no inflammation; 1, mild; 2, moderate; 3, severe), portal inflammation (0, no inflammation; 1, mild; 2, moderate; 3, severe), and necrosis (0, no necrosis; 1, ˂10% of liver parenchyma; 2, 10%–25% of liver parenchyma; 3, ˃25% of liver parenchyma) were scored and summed up to determine the overall histopathology score. Six 3 μm sections from each treatment group were scored by a trained, blinded researcher.

### Antioxidant capacity

Serum samples were used to assay total antioxidant capacity (T-AOC, #A015-2-1) and malondialdehyde (MDA, #A003-1) concentrations. The activities of the antioxidant enzymes superoxide dismutase (SOD, #A001-3), glutathione peroxidase (GSH-Px, #A005-1-2), and catalase (CAT, #A007-1-1) were measured using commercial assay kits (Nanjing Jiancheng, Institute of Bioengineering, Nanjing, China) according to the manufacturer’s instructions.

One Gram of liver tissue preserved at −80°C was homogenized with 9 mL of 0.9% ice-cold sodium chloride buffer and then centrifuged at 4,000 × g for 10 min at 4°C, and the supernatant was collected and analyzed immediately. The total antioxidant capacity (T-AOC, #A015-1-2) and activities of superoxide dismutase (T-SOD, #A001-1-2), glutathione peroxidase (GSH-Px, #A005-1-2), and catalase (CAT, #A007-1-1), as well as malondialdehyde (MDA, #A003-1) content, were measured. All readings were obtained using a microplate reader (Synergy H1; BioTek, Winooski, VT, United States).

### Serum stress indices and inflammatory factors

The concentrations of corticosterone, endotoxin, IL-1β, TNF-α, IL-6, and IL-10 in serum samples were measured using chicken-specific quantification ELISA kits purchased from Shanghai Enzyme-linked Biotechnology Co., Ltd. (Shanghai, China).

### Quantification of messenger ribonucleic acid (mRNA) by real-time PCR

Total RNA from liver samples (about 50–100 mg) was extracted by adding 1 mL of TRIzol-regent (#R601-03; TaKaRa Biotechnology, Dalian, Liaoning, China) according to the manufacturer’s instructions. The concentration and purity of total RNA were assessed using a spectrophotometer (NanoDrop Technologies, Wilmington, DE, United States). Subsequently, the RNA was reverse transcribed to cDNA using the PrimeScripte™ RT reagent Kit (Perfect Real Time, SYBR^®^ PrimeScriP™ TaKaRa, China, #3894A) according to the manufacturer’s instructions. The cDNA samples were amplified by qRT-PCR using the SYBR Premix Ex Taq reagent (Takara Biotechnology). The real-time PCR cycling conditions were as follows: 95°C for 30 s, 40 cycles of 95°C for 5 s, and 60°C for 30 s. The mRNA expression of target genes relative to beta-actin (β-actin) was calculated using the 2^−ΔΔCT^ method (Livak and Schmittgen, 2001). All the primer sequences are listed in Table 2 and were synthesized by Sangon Biotech Co., Ltd. (Shanghai, China).

**TABLE 2:** Primer sequences used for quantitative real-time PCR.

Genes	Primers sequence (5′-3′)	Accession number
Nrf2	F: TTC​GCA​GAG​CAC​AGA​TAC​TTC	NM_205117.1
	R: TGG​GTG​GCT​GAG​TTT​GAT​TAG	
Keap1	F: CTG​CTG​GAG​TTC​GCC​TAC​AC	XM_025145847.1
	R: CAC​GCT​GTC​GAT​CTG​GTA​CA	
SOD	F: GGT​CAT​CCA​CTT​CCA​GCA​GCA​G	U28407.1
	R: TAA​ACG​AGG​TCC​AGC​ATT​TCC​AGT​TAG	
CAT	F: TTG​CTA​TAC​GGT​TCT​CCA​CTG​TTG​C	NM_001031215.2
	R: GTA​AAG​ACT​CAG​GGC​GAA​GAC​TCA​AG	
GSH-Px	F: GAC​CAA​CCC​GCA​GTA​CAT​CA	NM_001277853.3
	R: GAG​GTG​CGG​GCT​TTC​CTT​TA	
β-Actin	F: GCT​ACA​GCT​TCA​CCA​CCA​CA	NM_205518.2
	R: TCT​CCT​GCT​CGA​AAT​CCA​GT	

Nrf2, nuclear factor erythroid 2-related factor 2; Keap1, Kelch-like ECH-associated protein 1; SOD, superoxide dismutase; CAT, catalase; GSH-Px, glutathione peroxidase; β-Actin, beta-actin.

### Statistical analysis

All data were preliminarily processed using the Excel 2010 software. Statistical analyses were performed using the statistical software SPSS 26.0, with replicates (*n* = 6) as an experimental unit, and the results are presented as mean ± SEM. Data were normally distributed (Shapiro-Wilk test) and evaluated for homogeneity of variance (Levene’s test). Statistical analyses were performed using one-way analysis of variance (ANOVA) followed by Tukey’s test. Differences were considered statistically significant at *p* < 0.05.

## Results

### Growth performance

During the LPS challenge (17–21 days of age), the ADG of broilers were decreased significantly compared with that of the CON group (*p* < 0.05). Moreover, the FCR of the MR1 group was significantly lower than that of the LPS group (*p* < 0.05). No significant difference was observed in the growth performance of broilers within the trial period (0–21 days of age), (*p* > 0.05) (Table 3).

**TABLE 3:** Effects of dietary methionine restriction on growth performance of LPS-stimulated broilers (n = 6).

Items^1^	17-21d			0-21d		
	ADFI/g	ADG/g	FCR	ADFI/g	ADG/g	FCR
CON	87.98 ± 0.09	57.13 ± 1.17^a^	1.54 ± 0.03^ab^	49.04 ± 1.43	32.55 ± 1.22	1.51 ± 0.01
LPS	78.16 ± 4.71	46.06 ± 2.04^b^	1.69 ± 0.03^a^	48.07 ± 0.79	31.43 ± 0.21	1.53 ± 0.03
MR1	77.87 ± 4.54	51.36 ± 3.05^ab^	1.52 ± 0.03^b^	44.07 ± 1.01	28.41 ± 1.22	1.55 ± 0.03
MR2	77.90 ± 3.40	50.79 ± 1.44^ab^	1.60 ± 0.07^ab^	46.06 ± 2.23	30.15 ± 1.27	1.53 ± 0.03
p-value	0.218	0.033	0.089	0.159	0.113	0.704

ADFI = average daily feed intake; ADG = average daily gain; FCR=feed conversion ratio.

^1^ CON, a basal diet plus intraperitoneal administration of sterile saline; LPS, a basal diet plus intraperitoneal administration of LPS; MR1, 0.3% mehtionine restriction diet plus intraperitoneal administration of LPS, MR2, 0.4% mehtionine restriction diet in combination with intraperitoneal administration of LPS.

^2^ Values were expressed as means ± SE.

^a-c^Means within a row with different superscripts are different at *p* < 0.05.

### Organ index

As shown in Table 4, at 3 h after injection, the spleen organ indices of broilers injected with LPS were increased (*p* < 0.05) and the thymus organ indices were decreased (*p* < 0.05) compared with the non-challenged broilers. The methionine-restricted diet significantly increased the thymus organ index (*p* < 0.05). The MR2 group showed a significantly increased in the spleen organ index (*p* < 0.05). At 8 h after injection, LPS challenge increased the spleen organ index (*p* < 0.05). The liver and bursa indices were not significantly different among the groups (*p* > 0.05).

**TABLE 4:** Effects of dietary methionine restriction on relative organ weigh of LPS-stimulated broilers (g/kg) (n = 6).

Items^1^	3 h after LPS stimulation				8 h after LPS stimulation			
	liver	bursa	spleen	thymus	liver	bursa	spleen	thymus
CON	30.82 ± 1.43	1.28 ± 0.10	0.86 ± 0.13^b^	3.11 ± 0.10^a^	28.03 ± 1.66	1.38 ± 0.11	0.90 ± 0.18^b^	2.61 ± 0.28
LPS	30.35 ± 1.02	1.43 ± 0.15	1.34 ± 0.05^a^	2.23 ± 0.28^b^	31.17 ± 0.88	1.64 ± 0.13	1.39 ± 0.17^a^	2.10 ± 0.29
MR1	35.27 ± 1.28	1.26 ± 0.16	1.09 ± 0.10^ab^	2.90 ± 0.33^a^	33.03 ± 1.36	1.79 ± 0.15	0.86 ± 0.04^ab^	2.59 ± 0.33
MR2	31.78 ± 1.21	1.47 ± 0.07	1.27 ± 0.15^a^	2.83 ± 0.22^a^	30.35 ± 0.42	1.45 ± 0.08	1.11 ± 0.14^ab^	2.83 ± 0.23
*p*-value	0.085	0.563	0.020	0.031	0.072	0.101	0.032	0.339

^1^ CON, a basal diet plus intraperitoneal administration of sterile saline; LPS, a basal diet plus intraperitoneal administration of LPS; MR1, 0.3% mehtionine restriction diet plus intraperitoneal administration of LPS, MR2, 0.4% mehtionine restriction diet in combination with intraperitoneal administration of LPS.

^2^ Values were expressed as means ± SE.

^a-c^Means within a row with different superscripts are different at *p* < 0.05.

### Serum biochemical parameters

As shown in Table 5, serum biochemical indicators did not differ significantly among the four groups at 3 h (*p* > 0.05). However, 8 h after the LPS injection, the serum biochemical indicators of AST and ALT were significantly higher in the LPS group than in the CON group (*p* < 0.05). In contrast, the MR1 group displayed significantly decreased AST activity and TP content compared with that of the LPS group (*p* < 0.05).

**TABLE 5:** Effects of dietary methionine restriction on serum biochemical parameters of LPS-stimulated broilers (n = 6).

Items^1^	3 h after LPS stimulation					8 h after LPS stimulation				
	ALT (ng/L)	AST (ng/L)	A/G	TP (g/L)	γ-GT (U/L)	ALT (ng/L)	AST (ng/L)	A/G	TP (g/L)	γ-GT (U/L)
CON	4.73 ± 0.46	301.13 ± 7.05	0.62 ± 0.03	27.52 ± 0.89	13.40 ± 0.75	3.43 ± 0.40^b^	268.78 ± 10.00^b^	0.61 ± 0.10	28.42 ± 1.11^a^	14.40 ± 2.24
LPS	4.30 ± 0.30	294.78 ± 14.17	0.61 ± 0.02	26.40 ± 1.56	13.52 ± 1.49	4.52 ± 0.54^a^	309.22 ± 9.96^a^	0.62 ± 0.02	28.97 ± 0.88^a^	12.5 ± 0.20
MR1	4.12 ± 0.30	294.43 ± 2.80	0.63 ± 0.02	24.10 ± 1.08	12.90 ± 0.84	43.5 ± 0.37^ab^	272.27 ± 8.05^b^	0.58 ± 0.02	24.73 ± 1.07^b^	11.97 ± 0.97
MR2	3.93 ± 0.34	301.37 ± 8.93	0.63 ± 0.03	24.05 ± 1.27	12.43 ± 1.05	4.45 ± 0.31^ab^	290.68 ± 7.91^ab^	0.61 ± 0.02	28.73 ± 1.38^b^	15.93 ± 1.40
*p*-value	0.448	0.912	0.955	0.149	0.885	0.089	0.018	0.956	0.047	0.208

ALT = alanine transaminase; AST = spartate transaminase; A:G = albumin to Globulin (A:G) ratio; TP = serum total protein; γ-GT = Gamma-glutamyl transpeptidase.

^1^ CON, a basal diet plus intraperitoneal administration of sterile saline; LPS, a basal diet plus intraperitoneal administration of LPS; MR1, 0.3% mehtionine restriction diet plus intraperitoneal administration of LPS, MR2, 0.4% mehtionine restriction diet in combination with intraperitoneal administration of LPS.

^2^ Values were expressed as means ± SE.

^a-c^Means within a row with different superscripts are different at *p* < 0.05.

### Liver damage

**FIGURE 1:**
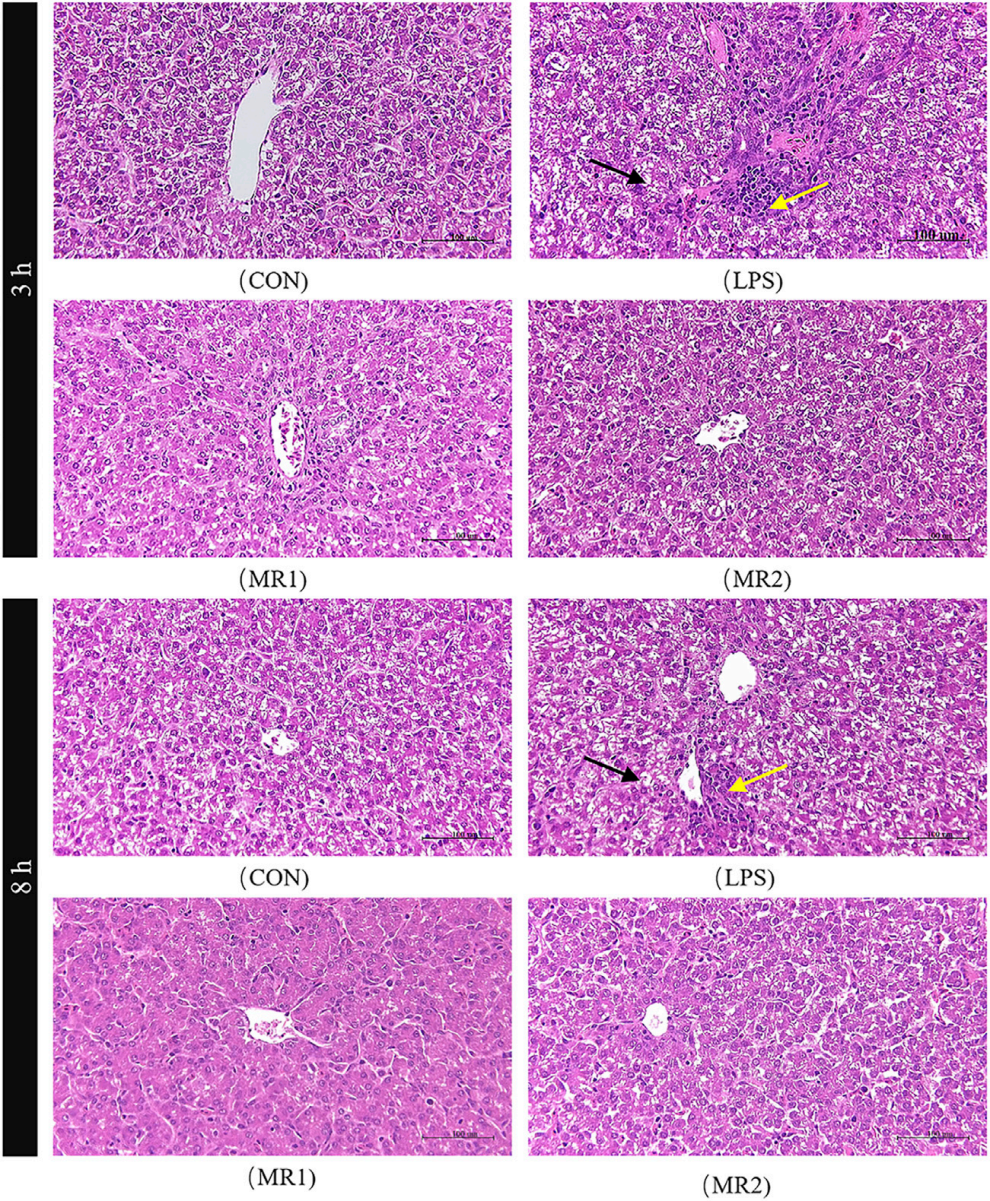
Effects of methionine restriction on histopathological analysis of liver in broilers stimulated by LPS (H and E staining, 400 ×). The yellow arrows point to inflammatory cells and the red scissors point to the vacuolar degeneration. CON, a basal diet plus intraperitoneal administration of sterile saline; LPS, a basal diet plus intraperitoneal administration of LPS; MR1, 0.3% mehtionine restriction diet plus intraperitoneal administration of LPS; MR2, 0.4% mehtionine restriction diet in combination with intraperitoneal administration of LPS. 3 h, 3 h after LPS stimulation; 8 h, 8 h after LPS stimulation.

**FIGURE 2:**
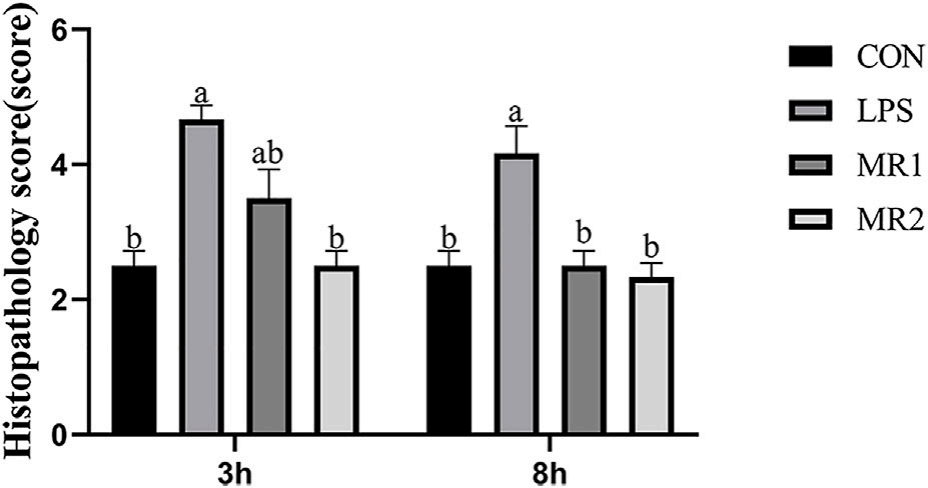
Effects of methionine restriction on histopathological score of liver in broilers stimulated by LPS. Data was shown as the mean ± SEM from six independent experiments. ^a–c^These bars without the same letter indicate differences significant at *p* < 0.05. CON, a basal diet plus intraperitoneal administration of sterile saline; LPS, a basal diet plus intraperitoneal administration of LPS; MR1, 0.3% mehtionine restriction diet plus intraperitoneal administration of LPS, MR2, 0.4% mehtionine restriction diet in combination with intraperitoneal administration of LPS. 3 h, 3 h after LPS stimulation; 8 h, 8 h after LPS stimulation.

Three hours after the LPS injection, the CON group presented with a normal liver architecture with little or no cell infiltration and minimal vacuolar degeneration, as well as neatly arranged hepatic cords. In LPS-challenged chickens, liver injury is characterized by infiltration of inflammatory cells such as heterophilic cells and lymphocytes, moderate and diffuse vacuolar degeneration, disorderly arrangement of hepatic cords, and more cells with nuclear fragmentation and necrosis. Compared with the LPS group, the hepatic cord structures of the MR1 and MR2 groups were more evident, no significant inflammatory cells were noted, and vacuolation degeneration was reduced (Figure 1). The assessment of liver sections from LPS-challenged chickens exhibited a significantly increased mean histopathology score of 4.67 ± 0.21, compared to a mean score of 2.50 ± 0.22 (*p* < 0.05) for the CON group. The MR2 diet showed significant suppression of acute liver injury with mean histopathology scores of 2.50 ± 0.22. The MR1 group also had a tendency to alleviate liver damage with scores of 3.50 ± 0.43 (Figure 2).

At 8 h, necrosis and moderate vacuolar degeneration were observed in the LPS group, around which different levels of inflammatory cell infiltration were observed. In comparison, the livers of the MR-treated chickens showed significant inhibition of the parameters of inflammatory damage and maintained normal liver architecture (Figure 1) (*p* < 0.05). Compared with a mean score of 2.50 ± 0.22 for the CON group, the LPS group showed a significantly increased mean histopathology score of 4.17 ± 0.41 (*p* < 0.05). However, MR1 and MR2 diets significantly reduced the degree of liver damage, shown in mean histopathology scores of 2.50 ± 0.22 and 2.33 ± 0.21 respectively (Figure 2) (*p* < 0.05).

### Liver and serum antioxidant capacity

At 3 h after LPS injection, the LPS challenge had no significant effect on liver antioxidant capacity (*p* > 0.05). However, the MR1 group significantly showed increased GSH-Px activity compared to that of the LPS group (*p* < 0.05). Lipopolysaccharide decreased T-AOC and CAT activity, and increased MDA content 8 h after LPS injection (*p* < 0.05). Compared to the LPS group, the MR1 group displayed significantly increased T-AOC levels and CAT, T-SOD, and GSH-Px activity, and decreased MDA content in the liver of broilers (*p* < 0.05). In addition, broilers in the MR2 group had higher CAT activity and lower MDA content than those in the LPS group (*p* < 0.05) (Table 6).

**TABLE 6:** Effects of dietary methionine restriction on liver antioxidant capacity of LPS-stimulated broilers (n = 6).

Items^1^	3 h after LPS stimulation					8 h after LPS stimulation				
	TAOC (U/mgprot)	MDA (nmol/mgprot)	CAT (U/mgprot)	T-SOD (U/mgprot)	GSH-Px (U/mgprot)	TAOC (U/mgprot)	MDA (nmol/mgprot)	CAT (U/mgprot)	T-SOD (U/mgprot)	GSH-Px (U/mgprot)
CON	1.60 ± 0.09	0.49 ± 0.05	10.12 ± 2.87	6.89 ± 0.35	42.11 ± 2.49^ab^	1.22 ± 0.13^a^	0.32 ± 0.05^b^	8.43 ± 0.36^a^	5.70 ± 0.42^b^	39.64 ± 5.28^ab^
LPS	1.87 ± 0.11	0.50 ± 0.04	5.02 ± 1.34	5.95 ± 0.48	37.29 ± 2.45^b^	0.67 ± 0.06^b^	0.72 ± 0.08^a^	6.28 ± 0.58^b^	5.85 ± 0.14^b^	30.67 ± 2.89^b^
MR1	1.60 ± 0.09	0.54 ± 0.06	10.03 ± 2.07	6.69 ± 0.59	47.86 ± 4.22^a^	1.16 ± 0.07^a^	0.40 ± 0.04^b^	8.51 ± 0.68^a^	8.16 ± 0.83^a^	43.84 ± 4.46^a^
MR2	1.86 ± 0.14	0.60 ± 0.10	7.14 ± 1.85	6.97 ± 0.61	37.19 ± 2.72^b^	0.94 ± 0.08^ab^	0.40 ± 0.09^b^	7.96 ± 0.48^a^	7.46 ± 0.42^ab^	38.57 ± 3.71^ab^
*p*-value	0.149	0.660	0.384	0.502	0.024	0.001	0.003	0.047	0.006	0.040

T-AOC = total antioxidant capacity; MDA = malonaldehyde; CAT = catalase; T-SOD = total superoxide dismutase; GSH-Px = glutathione peroxidase.

^1^ CON, a basal diet plus intraperitoneal administration of sterile saline; LPS, a basal diet plus intraperitoneal administration of LPS; MR1, 0.3% mehtionine restriction diet plus intraperitoneal administration of LPS, MR2, 0.4% mehtionine restriction diet in combination with intraperitoneal administration of LPS.

^2^ Values were expressed as means ± SE.

^a-c^Means within a row with different superscripts are different at *p* < 0.05.

In the case of serum, LPS treatment significantly decreased T-AOC, SOD, and GSH-Px activity at 3 h after LPS injection (*p* < 0.05). However, broilers in the MR1 group had higher CAT and SOD activities and T-AOC levels than those in the LPS group (*p* < 0.05). In addition, broilers in the MR2 group had higher SOD activity and T-AOC levels than those in the LPS group (*p* < 0.05). At 8 h after stimulation, LPS significantly increased (*p* < 0.05) MDA content and decreased (*p* < 0.05) T-AOC and GSH-Px activity compared with that of the CON group. The MR1 group showed significantly increased T-AOC and SOD activity but decreased MDA content compared to that of the LPS group (*p* < 0.05). Meanwhile, the MR2 group showed significantly increased T-AOC and decreased MDA content (*p* < 0.05) compared to that of the LPS group (Table 7).

**TABLE 7:** Effects of dietary methionine restriction on serum antioxidant capacity of LPS-stimulated broilers (n = 6).

Items^1^	3 h after LPS stimulation					8 h after LPS stimulation				
	T-AOC (U/mL)	MDA (nM)	CAT (ng/L)	SOD (pg/mL )	GSH-Px (IU/L)	T-AOC (U/mL)	MDA (nM)	CAT (ng/L)	SOD (pg/mL)	GSH-Px (IU/L)
CON	1.44 ± 0.03^a^	2.34 ± 0.19	2.29 ± 0.28^ab^	110.29 ± 4.59^a^	311.39 ± 8.96^a^	1.63 ± 0.05^a^	2.34 ± 0.19^b^	2.20 ± 0.21	104.22 ± 19.33^ab^	340.18 ± 7.01^a^
LPS	1.21 ± 0.05^b^	2.66 ± 0.18	1.44 ± 0.34^b^	80.07 ± 3.70^b^	258.22 ± 7.37^b^	1.36 ± 0.05^b^	2.84 ± 0.17^a^	2.20 ± 0.24	92.86 ± 5.26^b^	270.73 ± 8.93^b^
MR1	1.55 ± 0.07^a^	2.75 ± 0.13	2.87 ± 0.33^a^	107.49 ± 6.26^a^	269.69± 4.44^b^	1.59 ± 0.04^a^	2.39 ± 0.11^b^	2.34 ± 0.18	119.59 ± 7.25^a^	265.61 ± 3.81^b^
MR2	1.56 ± 0.05^a^	2.39 ± 0.09	1.75 ± 0.35^ab^	117.86 ± 5.35^a^	259.88 ± 2.45^b^	1.64 ± 0.05^a^	2.39 ± 0.09^b^	2.80 ± 0.34	116.67 ± 8.97^ab^	269.43 ± 5.96^b^
*p*-value	0.001	0.203	0.029	0.001	<0.001	0.001	0.035	0.598	0.036	<0.001

T-AOC = total antioxidant capacity; MDA = malonaldehyde; CAT = catalase; T-SOD = total superoxide dismutase; GSH-Px = glutathione peroxidase.

^1^ CON, a basal diet plus intraperitoneal administration of sterile saline; LPS, a basal diet plus intraperitoneal administration of LPS; MR1, 0.3% mehtionine restriction diet plus intraperitoneal administration of LPS, MR2, 0.4% mehtionine restriction diet in combination with intraperitoneal administration of LPS.

^2^ Values were expressed as means ± SE.

^a-c^Means within a row with different superscripts are different at *p* < 0.05.

### Serum stress indexes and pro-inflammation factors

As showen in Table 8, the LPS group had a higher serum LPS, CORT, IL-1β, IL-6, and TNF-αcontent, but lower levels of IL-10 than the CON group at 3 h and 8 h after LPS stimulation (*p* < 0.05). Meanwhile, MR groups (MR1 and MR2) significantly decreased LPS, CORT, IL-1β, IL-6, and TNF-α, but increased IL-10 contents compared with the LPS group at both time points (*p* < 0.05).

**TABLE 8:** Effects of dietary methionine restriction on serum stress indexes and pro-inflammation factors of LPS-stimulated broilers (n = 6).

Items^1^	3 h after LPS stimulation						8 h after LPS stimulation					
	LPS (ng/L)	CORT (ng/L)	IL-1β (ng/L)	IL-6 (ng/L)	TNF-α (ng/L)	IL-10 (ng/L)	LPS (ng/L)	CORT (ng/L)	IL-1β (ng/L)	IL-6 (ng/L)	TNF-α (ng/L)	IL-10 (ng/L)
CON	258.15 ± 2.71^b^	418.01 ± 6.26^b^	130.44 ± 2.46^b^	50.55 ± 0.98^b^	68.45 ± 1.55^b^	61.72 ± 1.12^b^	269.02 ± 6.05^b^	423.22 ± 5.08^b^	136.72 ± 3.22^b^	52.63 ± 0.77^b^	71.58 ± 1.35^b^	57.30 ± 0.66^b^
LPS	292.29 ± 4.82^a^	466.36 ± 10.37^a^	145.42 ± 2.82^a^	59.59 ± 0.97^a^	78.73 ± 1.79^a^	48.71 ± 1.36^c^	305.89 ± 2.57^a^	484.38 ± 7.79^a^	155.28 ± 1.16^a^	63.60 ± 1.79^a^	81.98 ± 0.61^a^	45.50 ± 1.99^c^
MR1	244.60 ± 4.60^b^	395.64 ± 8.47^bc^	126.19 ± 2.04^b^	47.88 ± 0.95^bc^	67.72 ± 1.50^b^	62.22 ± 1.16^ab^	253.24 ± 4.91^b^	413.72 ± 6.54^b^	132.31 ± 2.76^b^	49.87 ± 0.57^bc^	67.57 ± 1.19^bc^	61.90 ± 1.02^ab^
MR2	224.89 ± 3.78^c^	372.46 ± 9.43^c^	121.17 ± 2.75^b^	44.58 ± 0.91^c^	64.68 ± 1.08^b^	67.18 ± 1.52^a^	253.72 ± 3.58^b^	384.88 ± 6.11^c^	131.65 ± 2.63^b^	48.95 ± 0.70^c^	64.81 ± 1.33^c^	63.62 ± 1.28^a^
*p*-value	<0.001	<0.001	<0.001	<0.001	<0.001	<0.001	<0.001	<0.001	0.001	<0.001	<0.001	<0.001

LPS = lipopolysaccharide; CORT = corticosterone; IL-1β = interleukin-1β; IL-6, = interleukin-6; TNF-α = tumour necrosis factor-α; IL-10 = interleukin-10.

^1^ CON, a basal diet plus intraperitoneal administration of sterile saline; LPS, a basal diet plus intraperitoneal administration of LPS; MR1, 0.3% mehtionine restriction diet plus intraperitoneal administration of LPS, MR2, 0.4% mehtionine restriction diet in combination with intraperitoneal administration of LPS.

### mRNA expression of antioxidant genes in the liver

At 3 h after injection, the level of *CAT* gene expression in the LPS group significantly decreased (*p* < 0.05). However, the MR1 group showed significantly increased expression of *Nrf2*, *CAT*, and *GSH-Px* genes (*p* < 0.05). In addition, the MR2 group showed a higher level of *CAT* gene expression (*p* < 0.05). At 8 h, the LPS group showed no significantly affected gene expression levels (*p* > 0.05), but the MR1 group showed significantly increased expression of *Nrf2*, *Keap1*, *SOD*, and *GSH-Px* (*p* < 0.05). Meanwhile, the MR2 group had higher expression of *Keap1*, *SOD*, and *GSH-Px* (*p* < 0.05) (Table 9).

**TABLE 9:** Effects of dietary methionine restriction on liver mRNA expression of LPS-stimulated broilers (n = 6).

	3 h after LPS stimulation					8 h after LPS stimulation				
Items^1^	*Nrf2*	*Keap1*	*CAT*	*SOD*	*GSH-Px*	*Nrf2*	*Keap1*	*CAT*	*SOD*	*GSH-Px*
CON	0.97 ±0.12^ab^	1.01 ± 0.07	1.00 ± 0.03^a^	1.07 ± 0.08^b^	1.05 ± 0.13^b^	1.15 ± 0.18^b^	1.24 ± 0.29^b^	1.06 ± 0.14	1.03 ± 0.13^ab^	1.02 ± 0.08^b^
LPS	0.73 ± 0.14^b^	0.85 ± 0.07	0.34 ± 0.02^c^	1.59 ± 0.02^ab^	0.70 ± 0.09^b^	0.85 ± 0.06^b^	1.15 ± 0.12^b^	0.82 ± 0.05	0.80 ± 0.03^b^	0.66 ± 0.02^b^
MR1	1.18 ± 0.24^a^	0.83 ± 0.04	0.60 ± 0.05^b^	2.16 ± 0.21^a^	1.77 ± 0.18^a^	3.32 ± 0.50^a^	3.35 ± 0.50^a^	0.75 ± 0.03	0.75 ± 0.03^a^	1.95 ± 0.31^a^
MR2	0.71 ± 0.09^b^	0.78 ± 0.08	0.60 ± 0.05^b^	2.09 ± 0.18^a^	0.99 ± 0.15^b^	2.04 ± 0.12^b^	4.38 ± 0.69^a^	0.87 ± 0.08	1.31 ± 0.05^a^	1.66 ± 0.12^a^
*p*-value	0.085	0.173	<0.001	0.006	<0.001	<0.001	0.004	0.138	<0.001	<0.001

*Nrf2* = nuclear factor-erythroid 2-related factor 2; *Keap1* = kelch-like ECH-associated protein l; *CAT* = catalase; *SOD* = superoxide dismutase; *GSH-Px* = glutathione peroxidase.

^1^ CON, a basal diet plus intraperitoneal administration of sterile saline; LPS, a basal diet plus intraperitoneal administration of LPS; MR1, 0.3% mehtionine restriction diet plus intraperitoneal administration of LPS, MR2, 0.4% mehtionine restriction diet in combination with intraperitoneal administration of LPS.

^2^ Values were expressed as means ± SE.

^a-c^Means within a row with different superscripts are different at *p* < 0.05.

## Discussion

Lipopolysaccharide-induced immune stress that leads to a decrease in the growth performance of broilers, mainly because the body’s protein anabolism is weakened, catabolism is enhanced, and the nutrients originally used for growth are transferred to resist the inflammatory reaction (Jiang et al., 2010; Yang et al., 2011). In the present study, LPS decreased the weight gain of broilers during the challenge, possibly due to a stress-related reduction in feed intake. The MR1 diet alleviated the negative effect of the LPS challenge on the FCR of broilers. This suggests that MR exerts a protective effect on the growth and development of broilers under stress. In addition, there was no significant difference between the growth performance of the MR groups and the control group, indicating that the growth performance of broilers would not be reduced by properly restricting of the dietary methionine levels correctly. Previous studies also have reported that appropriate MR does not lead to weight loss (Thivat et al., 2009).

The thymus is the site of differentiation, development, and maturation of T-cells, and thymus enlargement is conducive to the enhancement of the cellular immune response in the body (Cooper et al., 2006). Our results revealed that the thymus index of the LPS group was decreased at 3 h, indicating that LPS could reduce cellular immune function in broilers. However, there was no difference in the thymus index between the LPS and control groups at 8 h, probably because the stimulation of LPS on the thymus was acute and subjects could recover quickly. The increase in thymus weight of LPS-challenged broilers on MR1 and MR2 diets indicated that the function of T lymphocytes and cellular immunity was enhanced. This might be attributed to the anti-inflammatory effects of MR.

Several studies have reported that LPS injection causes liver damage and dysfunction in broilers (Morris et al., 2014; Jangra et al., 2020; Mei et al., 2020). Our pathological observation and scoring of the liver at two different time intervals showed that LPS could cause liver damage, while it could be alleviated by both types of MR diets. In addition, AST and ALT activities in serum are used clinical indicators of liver injury (Senior, 2012). Our results showed that at 8 h, the serum AST and ALT activities of broilers in the LPS group were higher than those in the CON group, which may be because of the time taken for transaminase in liver cells to enter the blood. However, the MR1 diet significantly decreased the AST elevation. Consistent with our results, Zhang et al. (2020) reported that endotoxins caused a significant increase in AST and ALT activities. The decrease in serum AST activity suggests that the MR1 diet could reduce LPS-induced liver injury. These results indicate that MR can protect the integrity of the broiler liver, and this protective effect may be attributed to the liver being the main organ for methionine metabolism. MR increases cystathionine β-synthase (CBS) and cystathionine *γ* cleaving enzyme (CSE) content in the liver, which promotes the synthesis of glutathione (GSH) and hydrogen sulphide (H_2_S) (Aggrey et al., 2018). Glutathione and H_2_S play antioxidant roles, thus protecting the liver from oxidative damage (Mou et al., 2020; Elwan et al., 2021).

Oxidative stress originates from metabolic disorders involving reactive oxygen species (ROS)/reactive nitrogen species production and antioxidant production (Surai et al., 2019). Endotoxin stimulation has been reported to increase ROS production, but it decreases the activity of antioxidant enzymes (Zheng et al., 2016; Ji et al., 2021). In birds, antioxidant enzymes, including SOD, CAT, and GSH-Px, play an important role in controlling the negative effects of oxidative stress and preventing further immunopathological damage to host tissues (Zheng et al., 2020). Consistent with previous reports, our data showed that CAT and T-AOC activities were inhibited in the LPS challenge group, but the MDA content in the liver was increased. Malondialdehyde is the final product of the peroxidation reaction of free radicals on lipids and the most common marker of oxidative stress (Kamboh et al., 2016). Interestingly, dietary MR tended to improve CAT activity and total antioxidant capacity and decrease MDA content. In addition, SOD and GSH-Px activities were higher in the MR groups than those in the control group. This indicates that a methionine-restricted diet can improve LPS-induced oxidative stress in the liver, which mainly occurring at 8 h after LPS stimulation. Previous studies have also showed that MR significantly decreases the levels of ROS and MDA, and increases the levels of GSH-Px and T-AOC in the liver of high-fat-fed mice, possibly because MR can reduce the production of mitochondrial ROS and increase the endogenous H_2_S sulfide to alleviate oxidative stress (Maddineni et al., 2013; Ying et al., 2015).

However, different results were observed for the serum. T-AOC, and SOD and GSH-Px activities were only reduced 3 h after LPS injection. After 8 h, besides the similar changes in T-AOC and GSH-Px, the MDA content in the serum was increased. This may be why MDA, as an end product of lipid peroxidation, takes time to be produced after LPS stimulation (Balabanlı and Balaban, 2015). In addition, compared with the liver, the antioxidant enzyme activity in the serum was altered 3 h after LPS stimulation. This may be because LPS is absorbed into the blood by the abdominal vein after intraperitoneal injection, which first affects the antioxidant enzyme activity in the blood, and then causes oxidative damage to the liver when the LPS in the blood is absorbed by the tissues (Suriguga et al., 2020). However, dietary methionine restriction alleviated the LPS-induced decline in T-AOC at both time intervals, suggesting that MR alleviates LPS-induced oxidative stress in serum. At 3 h after stimulation, the MR1 diet alleviated the decrease in SOD and CAT activities caused by LPS, while the MR2 diet mainly alleviated SOD activity. After 8 h, both MR diets alleviated the increase in MDA content induced by LPS, and the MR1 diet improved the activity of SOD. Wu et al. (2020b) also showed that MR could increase serum T-AOC levels and SOD activity and reduce serum MDA levels in mice fed a high-fat diet. These results indicate that a methionine-restricted diet may increase the antioxidant capacity of broilers by increasing the activity of antioxidant enzymes, possibly owing the activation of antioxidant pathway gene expression.

Lipopolysaccharide causes oxidative stress and promotes inflammatory reactions (Rosadini and Kagan, 2017). It has been shown to increase the secretion of proinflammatory cytokines and inhibit animal growth (Takahashi, 2012; Tan et al., 2014; Li et al., 2015). Our results revealed that LPS stimulation significantly increased serum inflammatory cytokines TNF-α, IL-1β, and IL-6, and reduced the concentration of anti-inflammatory cytokine IL-10 at both time intervals. This indicates that the inflammation model was successfully established. However, MR diets significantly reduced the concentration of pro-inflammatory cytokines and increased the concentration of IL-10. A moderate reduction in dietary methionine level may alleviate LPS-induced inflammation. Zhang et al. (2019) discovered that MR significantly reduced plasma LPS and LBP concentrations in mice fed a high-fat diet and reduced the mRNA expression of the ileum genes TNF-αand IL-6, indicating that MR can reduce the inflammatory response by limiting the expression of inflammatory factors. In addition, Wu et al. (2020b) showed that MR could improve the intestinal flora, reduce the serum LPS concentration, and enhance the intestinal barrier of mice by upregulating single-chain fatty acid-producing bacteria and downregulating inflammatory LPS-producing bacteria. Our research also showed that MR could reduce the LPS concentration in the serum during the study period, and strengthen the intestinal immune barrier which might inhibit inflammation. Both oxidative stress and inflammation are considered to be the key factors of acute bacterial disease, and they promote and amplify each other. For example, the pro-inflammatory master gene NF-κB is a redox sensitive transcription factor. Conversely, NF-κB activates the genes of NADPH oxidase and COX2 (Arioz et al., 2019; Yu et al., 2022). The inhibitory effect of MR on inflammation and its direct antioxidant effect can break this vicious cycle.

*Nrf2* is a major regulator of cell defense against oxidative stress and is activated to promote the expression of antioxidant genes; *Keap1*, as an adaptor subunit of Cullin 3-based E3 ubiquitin ligase, regulates Nrf2 activity (Sajadimajd and Khazaei, 2018). The results showed that MR1 increased the gene expression of *Nrf2* at 3 h and 8 h, while MR1 and MR2 increased the gene expression of *Keap1* at 8 h alone. Previous studies have shown that during MR, the activity of the methionine metabolizing enzyme CBS and CSE was upregulated, which led to an increase in H_2_S and other downstream effectors (such as GSH and related compounds) (McIsaac et al., 2012; Petti et al., 2012). It has been observed that H_2_S modifies proteins post-translationally through S-sulfhydrination, transforming the—SH of cysteine into—SH, thus regulating protein activity. S-thinning of Kelch-like ECH-related protein 1 (*Keap1*) can activate *Nrf2* (Yang et al., 2013). Methionine restriction may directly activate *Nrf2* (Lewis et al., 2010). The increase in *Nrf2* activity and the accompanying enhanced gene expression of antioxidant response elements will improve the antioxidant and detoxification capacities of the body. Our results suggest that the MR1 diet activated the *Nrf2* pathway, and increased the gene expression of *SOD*, *CAT*, and *GSH-PX* at 3 h and 8 h after LPS injection. However, compared with MR1, the activation of the antioxidant pathway rate with the MR2 diet was slower until 8 h after injection.

## Conclusion

In conclusion, appropriately reducing methionine levels in the diet ameliorated LPS-induced immune stress and liver damage by inhibiting inflammation and oxidative stress. In addition, reducing methionine in the diet by 0.1% and 0.2% had a similar effect on relieving stress when injecting LPS at different times; however, MR1 activated the Nrf2 pathway earlier than MR2. The results confirmed the immune regulation, anti-oxidation, and anti-inflammatory effects of two different levels of MR diets on LPS-challenged broilers under high stocking density, and provided a scientific basis for the actual production of broilers.

## Data Availability

The original contributions presented in the study are included in the article/supplementary materials, further inquiries can be directed to the corresponding author.

## References

[B1] Aggrey S. E. Gonzalez-Ceron F. Rekaya R. Mercier Y. (2018). Gene expression differences in the methionine remethylation and transsulphuration pathways under methionine restriction and recovery with D,L-methionine or D,L-HMTBA in meat-type chickens. J. Anim. Physiol. Anim. Nutr. Berl. 102 (1), e468–e475. 10.1111/jpn.12779 28984387

[B2] Arioz B. I. Tastan B. Tarakcioglu E. Tufekci K. U. Olcum M. Ersoy N. (2019). Melatonin attenuates LPS-induced acute depressive-like behaviors and microglial NLRP3 inflammasome activation through the SIRT1/nrf2 pathway. Front. Immunol. 10, 1511. 10.3389/fimmu.2019.01511 31327964 PMC6615259

[B3] Averós X. Estevez I. (2018). Meta-analysis of the effects of intensive rearing environments on the performance and welfare of broiler chickens. Poult. Sci. 97 (11), 3767–3785. 10.3382/ps/pey243 29924356 PMC6162358

[B4] Balabanlı B. Balaban T. (2015). Investigation into the effects of boron on liver tissue protein carbonyl, MDA, and glutathione levels in endotoxemia. Biol. Trace Elem. Res. 167 (2), 259–263. 10.1007/s12011-015-0301-z 25787825

[B5] Brown-Borg H. M. Buffenstein R. (2017). Cutting back on the essentials: Can manipulating intake of specific amino acids modulate health and lifespan? Ageing Res. Rev. 39, 87–95. 10.1016/j.arr.2016.08.007 27570078 PMC5571732

[B6] Bryant C. E. Spring D. R. Gangloff M. Gay N. J. (2010). The molecular basis of the host response to lipopolysaccharide. Nat. Rev. Microbiol. 8 (1), 8–14. 10.1038/nrmicro2266 19946286

[B7] Cooper M. D. Peterson R. D. South M. A. Good R. A. (2006). The functions of the thymus system and the bursa system in the chicken. J. Immunol. 176 (11), 6370–6404. 16709795

[B8] Deng J. Zhang J. Chang Y. Wang S. Shi M. Miao Z. (2022). Effects of Chinese yam polysaccharides on the immune function and serum biochemical indexes of broilers. Front. Vet. Sci. 9, 1013888. 10.3389/fvets.2022.1013888 36148469 PMC9485930

[B9] Duan J. Xiang L. Yang Z. Chen L. Gu J. Lu K. (2022). Methionine restriction prevents lipopolysaccharide-induced acute lung injury via modulating CSE/H2S pathway. Nutrients 14 (2), 322. 10.3390/nu14020322 35057502 PMC8777780

[B10] Elwan H. Xie C. Miao L. P. Dong X. Zou X. T. Mohany M. (2021). Methionine alleviates aflatoxinb1-induced broiler chicks embryotoxicity through inhibition of caspase-dependent apoptosis and enhancement of cellular antioxidant status. Poult. Sci. 100 (8), 101103. 10.1016/j.psj.2021.101103 34229218 PMC8261005

[B11] Fang H. Stone K. P. Wanders D. Forney L. A. Gettys T. W. (2022). The origins, evolution, and future of dietary methionine restriction. Annu. Rev. Nutr. 42, 201–226. 10.1146/annurev-nutr-062320-111849 35588443 PMC9936953

[B12] Goo D. Kim J. H. Park G. H. Delos Reyes J. B. Kil D. Y. (2019). Effect of heat stress and stocking density onGrowth performance, breast meat Quality,and intestinal barrier function in broiler chickens. Anim. (Basel) 9 (3), 107. 10.3390/ani9030107 PMC6466317 30901977

[B13] Jangra A. Rajput P. Dwivedi D. K. Lahkar M. (2020). Amelioration of repeated restraint stress-induced behavioral deficits and hippocampal anomalies with taurine treatment in mice. Neurochem. Res. 45 (4), 731–740. 10.1007/s11064-019-02945-8 31898086

[B14] Ji S. Y. Cha H. J. Molagoda I. M. N. Kim M. Y. Kim S. Y. Hwangbo H. (2021). Suppression of lipopolysaccharide-induced inflammatory and oxidative response by 5-aminolevulinic acid in RAW 264.7 macrophages and zebrafish larvae. Biomol. Ther. Seoul. 29 (6), 685–696. 10.4062/biomolther.2021.030 33820881 PMC8551728

[B15] Jiang Z. Schatzmayr G. Mohnl M. Applegate T. J. (2010). Net effect of an acute phase response-partial alleviation with probiotic supplementation. Poult. Sci. 89 (1), 28–33. 10.3382/ps.2009-00464 20008799

[B16] Jin S. W. Zhang L. Lian Q. Q. Yao S. L. Wu P. Zhou X. Y. (2006). Close functional coupling between Ca^2+^ release-activated Ca^2+^ channels and reactive oxygen species production in murine macrophages. Mediat. Inflamm. 2006 (6), 36192. 10.1155/mi/2006/36192 PMC1775034 17392583

[B17] Kamboh A. A. Hang S. Q. Khan M. A. Zhu W. Y. (2016). *In vivo* immunomodulatory effects of plant flavonoids in lipopolysaccharide-challenged broilers. Animal 10 (10), 1619–1625. 10.1017/S1751731116000562 27079952

[B18] Leshchinsky T. V. Klasing K. C. (2001). Divergence of the inflammatory response in two types of chickens. Dev. Comp. Immunol. 25 (7), 629–638. 10.1016/S0145-305X(01)00023-4 11472784

[B19] Lewis K. N. Mele J. Hayes J. D. Buffenstein R. (2010). Nrf2, a guardian of healthspan and gatekeeper of species longevity. Integr. Comp. Biol. 50 (5), 829–843. 10.1093/icb/icq034 21031035 PMC2965188

[B20] Li M. Chen L. Zhao Y. Sun H. Zhao L. (2022). Research on the mechanism of HRP relieving IPEC-J2 cells immunological stress based on transcriptome sequencing analysis. Front. Nutr. 9, 944390. 10.3389/fnut.2022.944390 35911118 PMC9336541

[B21] Li Y. Zhang H. Chen Y. P. Yang M. X. Zhang L. L. Lu Z. X. (2015). Bacillus amyloliquefaciens supplementation alleviates immunological stress in lipopolysaccharide-challenged broilers at early age. Poult. Sci. 94 (7), 1504–1511. 10.3382/ps/pev124 26009750

[B22] Livak K. J. Schmittgen T. D. (2001). Analysis of relative gene expression data using real-time quantitative PCR and the 2(-Delta Delta C(T)) Method. Methods 25 (4), 402–408. 10.1006/meth.2001.1262 11846609

[B23] Ma B. Xing T. Li J. Zhang L. Jiang Y. Gao F. (2022). Chronic heat stress causes liver damage via endoplasmic reticulum stress-induced apoptosis in broilers. Poult. Sci. 101 (10), 102063. 10.1016/j.psj.2022.102063 36049294 PMC9445382

[B24] Maddineni S. Nichenametla S. Sinha R. Wilson R. P. Richie J. P. Jr. (2013). Methionine restriction affects oxidative stress and glutathione-related redox pathways in the rat. Exp. Biol. Med. (Maywood) 238 (4), 392–399. 10.1177/1535370213477988 23760005

[B25] McIsaac R. S. Petti A. A. Bussemaker H. J. Botstein D. (2012). Perturbation-based analysis and modeling of combinatorial regulation in the yeast sulfur assimilation pathway. Mol. Biol. Cell 23 (15), 2993–3007. 10.1091/mbc.E12-03-0232 22696683 PMC3408425

[B26] Mei W. Hao Y. Xie H. Ni Y. Zhao R. (2020). Hepatic inflammatory response to exogenous LPS challenge is exacerbated in broilers with fatty liver disease. Anim. (Basel) 10 (3), 514. 10.3390/ani10030514 PMC7143745 32204385

[B27] Morris A. Shanmugasundaram R. Lilburn M. S. Selvaraj R. K. (2014). 25-Hydroxycholecalciferol supplementation improves growth performance and decreases inflammation during an experimental lipopolysaccharide injection1 1Author disclosures: This research was partially supported by Dutch State Mines (DSM, Heerlen, The Netherlands) and Hatch grant awarded to RKS. Poult. Sci. 93 (8), 1951–1956. 10.3382/ps.2014-03939 24931970

[B28] Mou Y. Wen S. Li Y. X. Gao X. X. Zhang X. Jiang Z. Y. (2020). Recent progress in Keap1-Nrf2 protein-protein interaction inhibitors. Eur. J. Med. Chem. 202, 112532. 10.1016/j.ejmech.2020.112532 32668381

[B29] Petti A. A. McIsaac R. S. Ho-Shing O. Bussemaker H. J. Botstein D. (2012). Combinatorial control of diverse metabolic and physiological functions by transcriptional regulators of the yeast sulfur assimilation pathway. Mol. Biol. Cell 23 (15), 3008–3024. 10.1091/mbc.E12-03-0233 22696679 PMC3408426

[B30] Rosadini C. V. Kagan J. C. (2017). Early innate immune responses to bacterial LPS. Curr. Opin. Immunol. 44, 14–19. 10.1016/j.coi.2016.10.005 27842237 PMC5426986

[B31] Sajadimajd S. Khazaei M. (2018). Oxidative stress and cancer: The role of Nrf2. Curr. Cancer Drug Targets 18 (6), 538–557. 10.2174/1568009617666171002144228 28969555

[B32] Senior J. R. (2012). Alanine aminotransferase: A clinical and regulatory tool for detecting liver injury–past, present, and future. Clin. Pharmacol. Ther. 92 (3), 332–339. 10.1038/clpt.2012.108 22871997

[B33] Siegmund B. Lear-Kaul K. C. Faggioni R. Fantuzzi G. (2002). Leptin deficiency, not obesity, protects mice from Con A-induced hepatitis. Eur. J. Immunol. 32 (2), 552–560. 10.1002/1521-4141(200202)32:2<552:Aid-immu552>3.0.Co;2-h 11828372

[B34] Surai P. F. Kochish II Fisinin V. I. Kidd M. T. (2019). Antioxidant defence systems and oxidative stress in poultry biology: An update. Antioxidants (Basel) 8 (7), 235. 10.3390/antiox8070235 31336672 PMC6680731

[B35] Suriguga S. Luangmonkong T. Mutsaers H. A. M. Groothuis G. M. M. Olinga P. (2020). Host microbiota dictates the proinflammatory impact of LPS in the murine liver. Toxicol Vitro 67, 104920. 10.1016/j.tiv.2020.104920 32590029

[B36] Takahashi K. (2012). Effect of a cultures of *Aspergillus oryzae* on inflammatory response and mRNA expression in intestinal immune-related mediators of male broiler chicks. J. Poult. Sci. 49 (2), 94–100. 10.2141/jpsa.011079

[B37] Takahashi K. Takimoto T. Sato K. Akiba Y. (2011). Effect of dietary supplementation of astaxanthin from Phaffia rhodozyma on lipopolysaccharide-induced early inflammatory responses in male broiler chickens (Gallus gallus) fed a corn-enriched diet. Anim. Sci. J. 82 (6), 753–758. 10.1111/j.1740-0929.2011.00898.x 22111631

[B38] Tan J. Liu S. Guo Y. Applegate T. J. Eicher S. D. (2014). Dietary L-arginine supplementation attenuates lipopolysaccharide-induced inflammatory response in broiler chickens. Br. J. Nutr. 111 (8), 1394–1404. 10.1017/s0007114513003863 24330949

[B39] Thivat E. Farges M. C. Bacin F. D'Incan M. Mouret-Reynier M. A. Cellarier E. (2009). Phase II trial of the association of a methionine-free diet with cystemustine therapy in melanoma and glioma. Anticancer Res. 29 (12), 5235–5240. 20044642

[B40] Wu G. Han L. Shi Y. Feng C. Yan B. Sun J. (2020a). Effect of different levels of dietary methionine restriction on relieving oxidative stress and behavioral deficits in middle-aged mice fed low-medium-or high-fat diet. J. Funct. Foods 65, 103782. 10.1016/j.jff.2020.103782

[B41] Wu G. Shi Y. Han L. Feng C. Ge Y. Yu Y. (2020b). Dietary methionine restriction ameliorated fat accumulation, systemic inflammation, and increased energy metabolism by altering gut microbiota in middle-aged mice administered different fat diets. J. Agric. Food Chem. 68 (29), 7745–7756. 10.1021/acs.jafc.0c02965 32597175

[B42] Xie M. Hou S. S. Huang W. Fan H. P. (2007). Effect of excess methionine and methionine hydroxy analogue on growth performance and plasma homocysteine of growing Pekin ducks. Poult. Sci. 86 (9), 1995–1999. 10.1093/ps/86.9.1995 17704389

[B43] Yang G. Zhao K. Ju Y. Mani S. Cao Q. Puukila S. (2013). Hydrogen sulfide protects against cellular senescence via S-sulfhydration of Keap1 and activation of Nrf2. Antioxid. Redox Signal 18 (15), 1906–1919. 10.1089/ars.2012.4645 23176571

[B44] Yang X. J. Li W. L. Feng Y. Yao J. H. (2011). Effects of immune stress on growth performance, immunity, and cecal microflora in chickens. Poult. Sci. 90 (12), 2740–2746. 10.3382/ps.2011-01591 22080012

[B45] Ying Y. Yun J. Guoyao W. Kaiji S. Zhaolai D. Zhenlong W. (2015). Dietary L-methionine restriction decreases oxidative stress in porcine liver mitochondria. Exp. Gerontol. 65, 35–41. 10.1016/j.exger.2015.03.004 25765145

[B46] Yu W. (2013). Modulation of tight junction protein expression in chicken intestine by methionine substitude and the mechanism. Beijing: Jiangnan University.

[B47] Yu Z. Zhao L. Zhao J.-L. Xu W. Guo Z. Zhang A.-Z. (2022). Dietary Taraxacum mongolicum polysaccharide ameliorates the growth, immune response, and antioxidant status in association with NF-κB, Nrf2 and TOR in Jian carp (*Cyprinus carpio* var. Jian). Aquaculture 547, 737522. 10.1016/j.aquaculture.2021.737522

[B48] Zhang Y. Jia H. Jin Y. Liu N. Chen J. Yang Y. (2020). Glycine attenuates LPS-induced apoptosis and inflammatory cell infiltration in mouse liver. J. Nutr. 150 (5), 1116–1125. 10.1093/jn/nxaa036 32101618

[B49] Zhang Y. Yang Y. Wang Y. (2019). Effect of methionine restriction on gut redox status, inflammation and microbiota in high-fat diet-fed mice. Food Sci. 40 (9), 99–106. 10.7506/spkx1002-6630-20171226-333

[B50] Zheng X. C. Wu Q. J. Song Z. H. Zhang H. Zhang J. F. Zhang L. L. (2016). Effects of Oridonin on growth performance and oxidative stress in broilers challenged with lipopolysaccharide. Poult. Sci. 95 (10), 2281–2289. 10.3382/ps/pew161 27143760

[B51] Zheng Y. W. Zhang J. Y. Zhou H. B. Guo Y. P. Ma Q. G. Ji C. (2020). Effects of dietary pyrroloquinoline quinone disodium supplementation on inflammatory responses, oxidative stress, and intestinal morphology in broiler chickens challenged with lipopolysaccharide. Poult. Sci. 99 (11), 5389–5398. 10.1016/j.psj.2020.08.007 33142455 PMC7647834

